# Histone Chaperone-Mediated Nucleosome Assembly Process

**DOI:** 10.1371/journal.pone.0115007

**Published:** 2015-01-22

**Authors:** Hsiu-Fang Fan, Zi-Ning Liu, Sih-Yao Chow, Yi-Han Lu, Hsin Li

**Affiliations:** 1 Department of Life Sciences and Institute of Genome Sciences, National Yang-Ming University, Taipei, Taiwan; 2 Biophotonics interdisciplinary research center, National Yang-Ming University, 112, Taipei, Taiwan; 3 Department of Biotechnology and Laboratory Science in Medicine, National Yang-Ming University, 112, Taipei, Taiwan; Ludwig-Maximilians-Universität München, GERMANY

## Abstract

A huge amount of information is stored in genomic DNA and this stored information resides inside the nucleus with the aid of chromosomal condensation factors. It has been reported that the repeat nucleosome core particle (NCP) consists of 147-*bp* of DNA and two copies of H2A, H2B, H3 and H4. Regulation of chromosomal structure is important to many processes inside the cell. *In vivo*, a group of histone chaperones facilitate and regulate nucleosome assembly. How NCPs are constructed with the aid of histone chaperones remains unclear. In this study, the histone chaperone-mediated nucleosome assembly process was investigated using single-molecule tethered particle motion (TPM) experiments. It was found that Asf1 is able to exert more influence than Nap1 and poly glutamate acid (PGA) on the nucleosome formation process, which highlights Asf1’s specific role in tetrasome formation. Thermodynamic parameters supported a model whereby energetically favored nucleosomal complexes compete with non-nucleosomal complexes. In addition, our kinetic findings propose the model that histone chaperones mediate nucleosome assembly along a path that leads to enthalpy-favored products with free histones as reaction substrates.

## Introduction

The nucleosome consists of ∼147-*bp* of genomic DNA, which is wrapped in two copies of H2A, H2B, H3 and H4 and consists of ∼1.7 left-handed helical turns [1–4]. This universal structure is responsible for the organization and compaction of DNA molecules in the nuclei of eukarya. It has been pointed out that the sequential assembly, governed by the intrinsic properties of the histone components, is at work *in vitro* [5–9]. So far, it has been reported that a tetrasome consisting of a H3/H4 tetramer and a DNA molecule is formed first and this is followed by the formation of the hexasome or nucleosome. It is well known that the dynamic regulation of nucleosomal structures *in vivo* plays an important role in gene expression and integrity maintenance[10]. Inside the cell, there are sophisticated systems that facilitate the proper organization of nucleosomal structures[11]. Histone chaperones are a group of acidic proteins that assist in histone trafficking, nucleosome assembly and nucleosome disassembly [12,13]. A range of distinct histone chaperones have been reported, including heterotrimeric chromatin assembly factor I (CAF1)[14,15], N1/N2[16], histone cell cycle regulation defective homolog A (HIRA)[17], nucleosome assembly protein 1(Nap1)[18,19] and anti-silencing factor 1(Asf1)[20,21]. Although these highly acidic proteins seem to have similar functions during the nucleosome assembly process, the structural motifs found within them seem to be unrelated [22–26]. For example, Nap1 is a homodimer, Asf1 is active as a monomer and NPM is a homo-pentamer in solution [23,24,27]. Some hetero-oligomeric chaperones have also been found, such as: FACT, which consists of SSRP1 and SPT16 [28].

Single molecule techniques have been used to study detailed molecular mechanisms that are inaccessible to conventional biochemical and biophysical approaches [29–38]. Recently, single-molecule techniques, including bead manipulation methods and fluorescence techniques, have been used to study the processes involved in histone chaperone-mediated nucleosome assembly/disassembly[39–44]. These studies found that histone chaperones not only affect the nucleosome assembly rate but also the nucleosome formation fraction [41,45]. Furthermore, a accumulated positive superhelical density in the range 0.025–0.051 will inhibit further nucleosome assembly[42]. A stepwise shortening in the DNA length of 50 nm has also been found [42,46]. Recently, Vlijm *et. al.* pointed out that a change in the linking number rather than a decrease in DNA length is solid evidence for the formation of intact nucleosomes [43]. Stepwise shortening in DNA length of ∼25 nm, which represents either the assembly of the H3/H4 tetrasome or the follow-up formation of the hexasome, has been observed[43]. Bennick *et. al.* applied force to a tethered DNA molecule in the presence of cell extract and found that nucleosome formation proceeds with a speed of 2∼3 nucleosome per second and then stalls when force is greater than 10 pN[47]. Wagner *et. al.* reported that a faster assembly rate and a higher nucleosome formation fraction can be observed for DNA molecules in response to cell extracts containing yNAP-1, which highlights the importance of histone chaperones[41]. Even though a potential mechanism for the histone chaperone-mediated nucleosome assembly process has been proposed based on the various thermodynamic constants already obtained[11], and a great deal of research is taking place in this area, the detailed assembly mechanism still remains controversial[5,8,11,41,42,44,48,49].

In the present study, single molecule tethered particle motion (TPM) was used to study the histone chaperone-mediated nucleosome assembly process *in vitro* in order to explore the detailed molecular mechanism involved. Compared to negatively-charged macromolecules, histone chaperones use not only electrostatic interactions but also substrate-specific hydrophobic interactions during histone-binding to facilitate nucleosome formation and prevent non-chromosomal interactions. That Asf1 plays more important role than Nap1 on the nucleosome assembly process was found, highlighting the substrate-specific interaction mode. Based on our kinetic results, it was found that a thermodynamically favored histone chaperone-histone complex is formed, rather than a ternary complex with a lower activation energy, when histone chaperone is present. Moreover, the reaction substrate during the histone chaperone-mediated nucleosome assembly process consists of free histones in dynamic equilibrium, rather than histone chaperone-histone complexes.

## Results

### Histone chaperone-mediated nucleosome assembly process investigated using tethered particle motion

**Figure 1:**
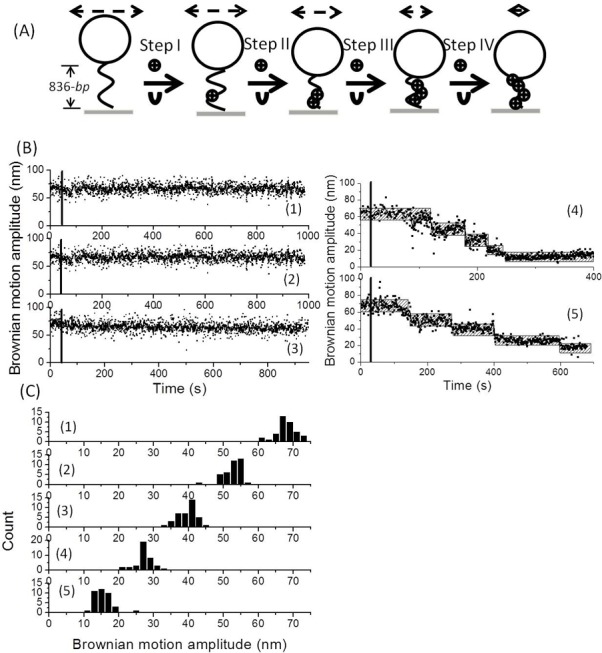
Tethered particle motion experiment investigating the histone chaperone-mediated nucleosome assembly process. (A) The schematic diagram illustrating the interaction between a 836-*bp* DNA molecule and histone in the presence of the histone chaperone, Nap1, allowing the formation of nucleosomes sequentially. The black wavy line denotes a DNA molecule anchored on a coverslip and attached to a streptavidin-labeled polystyrene bead (200 nm in diameter; drawn as a sphere) at the other. The circle denotes the histone components (H2A/H2B dimer, H3/H4 tetramer or histone octamer). The U-shaped figure denotes Nap1. The dash double-arrowed line represents the Brownian motion amplitude of tethered molecule. (B) A representative time trace for the DNA molecule in response to the addition of (i) buffer, (ii) 15 nM Nap1, (iii) 7.5 nM H2A/H2B dimer along with15 nM Nap1, (iv) 7.5 nM H3/H4 tetramer along with 15 nM Nap1, and (v) 7.5 nM histone octamer along with15nMNap1. The black line indicates the initiation of reaction. The hatched bar represents the expected BM amplitude for a 836-*bp* molecule in response to the addition of histone, which then forms the tetrasome or nucleosome. (C) The distribution of Brownian motion amplitude for the 836-*bp* DNA molecule in response to the addition of histone octamer along with Nap1 obtained from various different time traces (N = 38). Average BM amplitude of 67.9±4.4 nm, 53.5±4.3 nm, 40.1±5.3 nm, 27.3±2.4 nm and 15.0±4.6 nm, were obtained and these correspond to the status (i)–v) depicted in Fig. 1(A).

A 836-*bp* DNA molecule was anchored onto the coverglass, while the topological change in the DNA molecule, as reflected in the BM amplitude of the tethered polystyrene bead, was monitored. A decrease in BM amplitude was expected and would signal the formation of nucleosomal structures (Fig. 1A). Histone chaperones are known to promote the formation of nucleosomal complexes. One well studied histone chaperone, Nap1, was chosen and investigated in detail in this study. No significant changes in the BM amplitude were observed with respect to the DNA molecule in response to the addition of reaction buffer and Nap1 only (Fig. 1B (1–2)). Even though it has been reported that the H2A/H2B dimer is able to bind to DNA molecules with a high affinity of 10∼50 nM, no detectable change in BM amplitude was found after addition of H2A/H2B dimer in the presence of Nap1 (Fig. 1B (3)); this is consistent with previous studies [43,49]. Next, a four-step decrease in the BM trajectory was observed once H3/H4 tetramer or histone octamer, in the presence of Nap1, was added into the reaction chamber (Fig. 1B (4–5)). The decrease in BM amplitude was the same at each step for both the H3/H4 tetramer and the histone octamer treatment, which indicates that TPM experiment is able to detect the first-step of nucleosome assembly process, namely the formation of tetrasome, but is unable to detect further assembly of H2A/H2B dimer. After analyzing the change in BM amplitude, an average BM step size of 12.5±4.8 nm between steps was found (Fig. 1C). In order to confirm that each step-wise decrease in BM amplitude represents the formation of one nucleosome, the same experiments were performed using DNA molecules with different lengths. One, two and three-step shortening in BM trajectory were found for the 211-*bp*, 433-*bp* and 614-*bp* DNA molecules respectively (S1 Fig.). This further supports the idea that each discreet decrease in BM trajectory with average BM step size of 12.5±4.8 nm signals the formation of nucleosome.

### The roles of histone chaperones in the nucleosome assembly process

**Figure 2:**
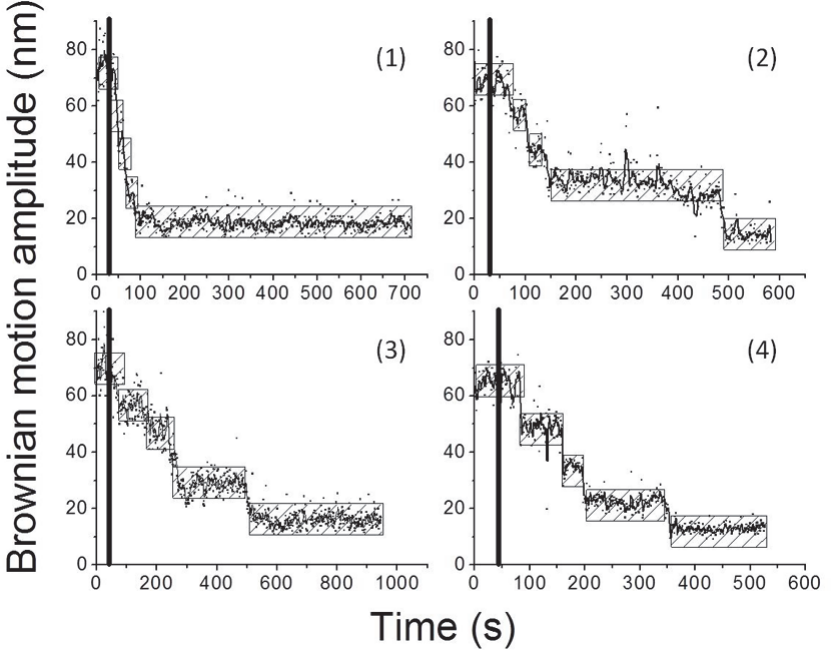
The representative time trace from the tethered particle motion experiments investigating the histone chaperone-mediated nucleosome assembly process. (A) 7.5 nM histone octamer only. (B) 7.5 nM histone octamer along with 15 nM Nap1. (C) 7.5 nM histone octamer along with 15 nM Asf1.(D) 7.5 nM histone octamer along with 37.5 nM PGA. The black line indicates the initiation of reaction. The hatched bar represents the expected BM amplitude for a 836-*bp* DNA molecule in response to the addition of histone to form nucleosomes. The assembly trace displays a four-step shortening in DNA length.

It was reported by Ruiz-Carillo *et. al.* that nucleosomes can be reconstituted successfully by the simultaneous addition of all core histones in the absence of histone chaperones[57]. Initially, the nucleosome assembly process was performed using a 836-*bp* DNA molecule in the absence of histone chaperones, and a representative BM trajectory is shown in Fig. 2 (1). A fast decrease in the BM amplitude after the addition of histone proteins was observed, indicating that there is shortening of the DNA’s length, which signals the formation of nucleosomes. However, it is also known that histone chaperones do bind histones and help to regulate the formation of nucleosome by preventing non-specific histone-DNA interactions[58]. Inside the cell, histone chaperones play a number of roles in various different steps of nucleosome assembly; these include histone import, histone transfer, histone supply and the deposition of nucleosome components [20,49,59,60]. Nap1 has an equal binding affinity toward the H3/H4 tetramer and the H2A/H2B dimer and is known to participate in the H2A/H2B dimer deposition [49,60]. Asf1 is involved in the formation, import and transfer of the H3/H4 tetramer [10,20].In order to check the influence of histone chaperones; two well known histone chaperones (Nap1 and Asf1) were chosen and investigated using TPM.

At first, control experiments were done only in the presence of histone chaperone (Nap1 and Asf1) or PGA, no significant change was observed (Fig. 1B (2) and S2 Fig.). A slower decrease in the BM trajectory was observed for DNA molecule in response to the addition of histone proteins in the presence of either Nap1 or Asf1 (Fig. 2 (2–3)). These results indicate that the rate of nucleosome assembly is reduced in the presence of these chaperones. Not only histone chaperones, but also negatively charged macromolecules, are known to facilitate nucleosome reconstitution in vitro[57]. In order to verify the effect of such electrostatic interactions, the same TPM experiments were performed in the presence of negatively charged macromolecules, namely PGA. A clear but slower four-step decrease in the BM trajectory was observed under this condition (Fig. 2 (4)). Interesting, the step size of the nucleosome assembly process was different when the treatments with histone chaperones and PGA were compared (S3 Fig.). A larger step size of 19.5±3.4 nm for the BM trajectory was found for the latter conditions, which reflects a more severe shortening in the DNA’s length (>147-*bp*). These findings imply that histone chaperones use not only electrostatic interactions but also specific modes of steric constraints and/or hydrophobic interactions in order to properly promote the correct chromosomal structures[20,61]

### Kinetic analysis of the histone chaperone-mediated nucleosome assembly process

**Figure 3:**
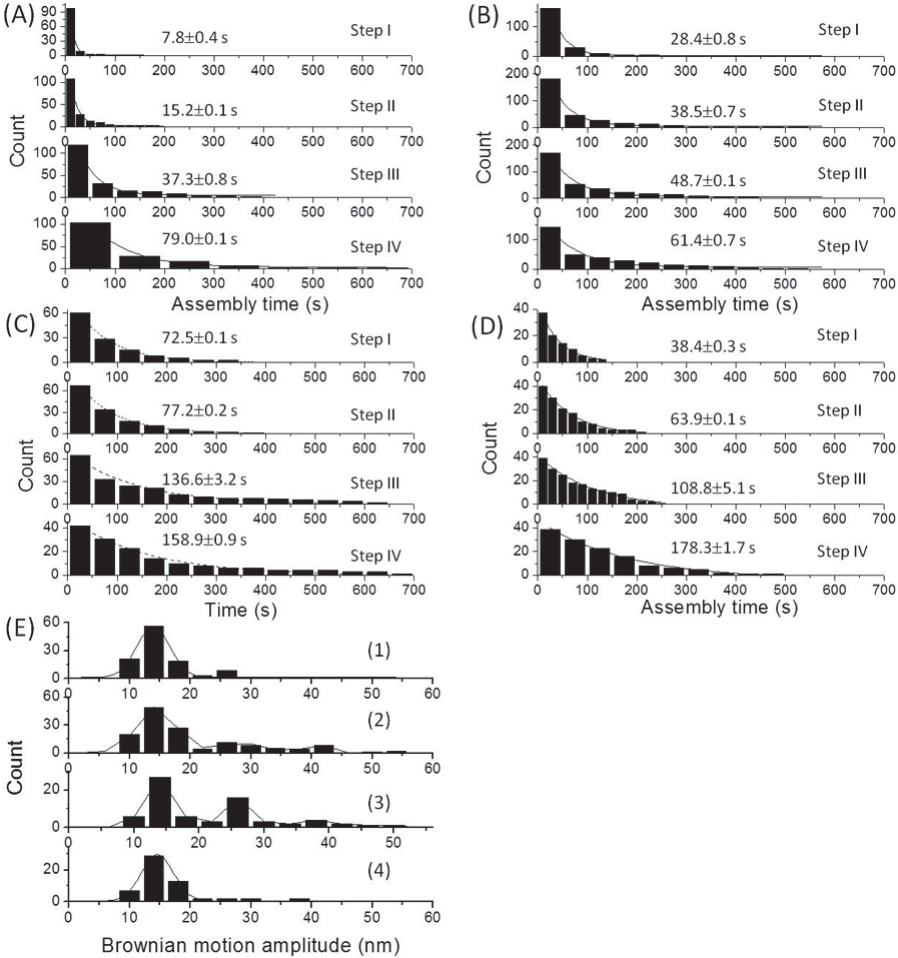
Kinetic analysis of the dwell times of the individual steps for a 836-bp DNA molecule in response to the histone octamer. The histograms were constructed from the pooled distribution of dwell times for the assembly state illustrated in Fig. 1A (i)–(iv). (A) histone octamer only. (B) histone octamer along with Nap1. (C) histone octamer along with Asf1. (D) histone octamer along with PGA. The time constants were obtained from single-exponential fitting using Origin 8.0. All the time constants are listed in Table 1. (E) The distribution of BM amplitude for 836-*bp* DNA molecules in response to the addition of histone octamer (i) no chaperone (N = 111). in the presence of (ii) Nap1 (N = 139). (iii) Asf1 (N = 89). (iv) PGA (N = 57).

Even though it has been pointed out that the histone chaperone-mediated nucleosome assembly process is reversible [49], a clear four-step decrease in the BM trajectory during the nucleosome assembly process was observed using TPM (Fig. 1B). In this context, sometimes in our experiments the dissociation of histones from DNA molecules was occasionally observed in the presence of histone chaperones (data not shown). Any DNA molecules that exhibited the dissociation of nucleosomes were excluded from the data analysis, and only the dwell time for the nucleosome assembly process was chosen and analyzed. Previously it has been pointed out that the rate limited step (RLS) of the nucleosome assembly process is the formation of the tetrasome, followed by the fast formation of the hexasome then the nucleosome[8,41]. Since the further addition of H2A/H2B dimer is undetectable in TPM experiments, the decrease in BM amplitude signals the deposit of H3/H4 tetramer onto DNA molecule to form the tetrasome[20,51]. Therefore, the dwell time of each BM amplitude state was used to describe the kinetics behavior of the first-step in the nucleosome assembly process, which will reflect the assembly time of tetrasome. The dwell times for the individual steps were pooled separately and fitted to a single exponential decay model (Fig. 3 A–D). For the histone only system, the dwell times for each assembly step were fitted to a single exponential model and this gave assembly times of (7.8±0.4) s, (15.2±0.1) s, (37.3±0.8) s and (79.0±0.1) s for first, second, third and fourth nucleosome assembly, respectively (Fig. 3A). Each assembly time is getting longer and longer, which is consistent with a previous report indicating that topological constrains inhibit the nucleosome assembly process[42]. In the presence of Nap1, assembly times of (28.4±0.8) s, (38.5±0.7) s, (48.7±0.5) s and (61.4±0.7) s were obtained (Fig. 3B), which reveals a much slower assembly process. This suggests that the histone chaperone is able to moderate the interactions between the histones and the DNA molecule. The same analysis of the nucleosome assembly process was performed in the presence of Asf1, and the assembly times obtained were (72.5±0.1) s, (77.2±0.2) s, (136.6±3.2) s and (158.9±0.9) s, which are all much longer than in the absence of histone chaperone (Fig. 3C). It would also seem that there is a more significant influence on the assembly time when Asf1 is present compared to Nap1. In addition, notwithstanding the fact that the BM step size in the nucleosome assembly process under PGA conditions showed different behavior, longer assembly times, of (38.4±0.3) s, (63.9±0.1) s, (108.8±5.1) s and (178.3±1.7) s were also obtained in the presence of PGA (Fig. 3D), indicating that PGA moderates the electrostatic interaction between the histones and the DNA molecule. Next the nucleosome formation fraction within a 15 minutes reaction time under different conditions was calculated, and a significantly smaller mucleosome formation fraction was obtained in the presence of the two histone chaperones (Fig. 3E and Table 1).

**Table 1:** Kinetic and thermodynamic information related to the histone chaperone-mediated nucleosome assembly process.

**Conditions**	**Histone (nM)**	**Chaperone (nM)**	**tAssembly, step I (s)**	**tAssembly, step II (s)**	**tAssembly, Step III (s)**	**tAssembly, step IV (s)**	**Package ratio (%, 15 min)**
No chaperone	7.5	15	7.8±0.4	15.2±0.1	37.3±0.8	79.0±0.1	87.3 (N = 111)
yNap1	7.5	15	28.4±0.8	38.5±0.7	48.7±0.1	61.4±0.7	70.5(N = 139)
yAsf1	7.5	15	72.5±0.1	77.2±0.2	136.6±3.2	158.9±0.9	40.4(N = 89)
PGA^a^	7.5.	15	38.4±0.3	63.9±0.1	108.8±5.1	178.3±1.7	87.7(N = 57)
yAsf1	7.5	37.5	143.4±9.5	133.7±3.6	149.0±7.8	155.9±17.5	19.4(N = 51)
yAsf1	7.5	75	424.23±86.30	181.76±8.73	N.D	N.D	<3.9(N = 31)

N.D: Not detectable

N: The total analyzed events or number of tethered DNA molecule

The interactions between nucleic acid molecules and proteins are highly ionic strength dependent. In order to confirm the importance of Asf1 in the first step of nucleosome assembly process, the same experiments were performed under low salt condition. A slower assembly process was observed both under histone only or under histone along with histone chaperones (Nap1 and Asf1) (S4A Fig.). Moreover, the assembly fraction for 836-bp DNA molecules in response to histone octamer with or without the aid of histone chaperone decreased under low salt condition (S4B Fig.) compared to that under 150 mM NaCl. This observation can be explained by the fact that the electrostatic repulsion between histone components is too strong and impedes the formation of H3/H4 tetramer and H2A/H2B dimer under lower ionic strength condition, highlighting the functional role of histone chaperones. A much slower assembly time was also observed when Asf1 is present compared to that of Nap1 (S4A Fig. (2–3), consistent to the results obtained under 150 mM NaCl. These observations reinforce that Asf1 plays more important role than Nap1 in the formation of tetrasomes.

**Figure 4:**
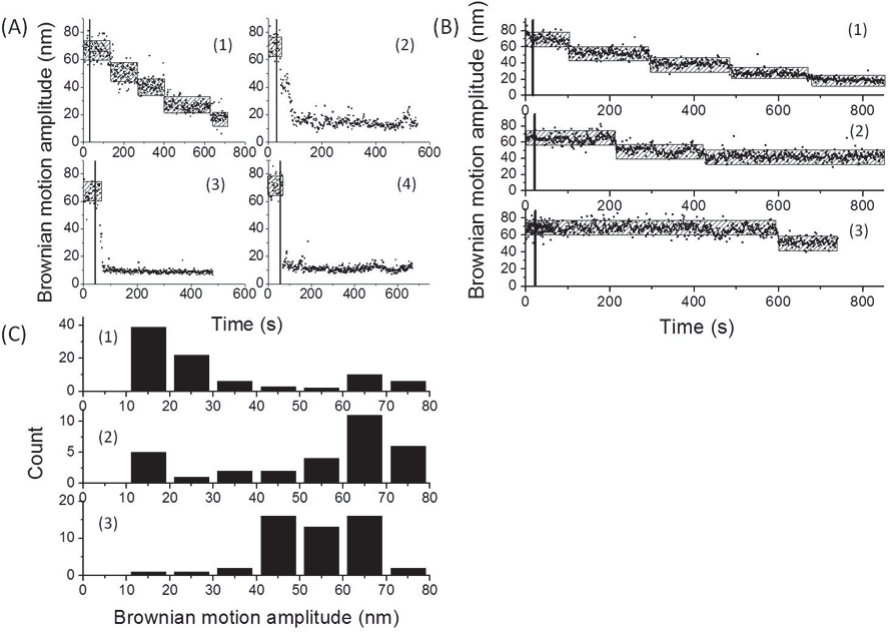
The effect of concentration on the histone chaperone-mediated nucleosome assembly process. (A) The BM time-trace of a 836-*bp* DNA molecule in response to the addition of 15 nM Asf1 along with different concentration of histone octamer (i) 7.5 nM. (ii) 15 nM. (iii) 30 nM. (iv) 60 nM. (B) The BM time-trace of a 836-*bp* DNA molecule in response to the addition of 7.5 nM histone octamer along with different concentration of yAsf1 (i) 15 nM. (ii) 37.5 nM. (iii) 75 nM. The black line indicates the initiation of the reaction. The hatched bar represents the expected BM amplitude for 836-bp DNA molecules in response to the addition of histone to form nucleosomes. (C) The distribution of BM amplitude for 836-*bp* DNA molecules in response to the addition of histone octamer along with different concentration of Asf1 (i) 15 nM (N = 89). (ii) 37.5 nM (N = 51). (iii) 75 nM (N = 31).

It remains unclear whether the substrate in the nucleosome assembly process is a chaperone-histone complex or the free histone molecules. In order to further clarify the mechanism of histone chaperone-mediated nucleosome assembly, the same TPM experiments and data analysis were performed using different concentration of histones and different concentrations of histone chaperones. The assembly process became too fast to be accurately determined when the concentration of histone molecules was raised (Fig. 4A). In contrast, the nucleosome assembly process became slower as the concentration of histone chaperones was raised (Fig. 4B and S5 Fig.), and a smaller nucleosome formation fraction was obtained within a 15 minutes reaction time once the histone chaperone concentration was raised (Fig. 4C and Table 1). These findings suggest that DNA molecules compete for the free histone in equilibrium with the chaperone-histone complex to form the nucleosomal complex.

## Discussion

Even though the interaction between H2A/H2B dimers and the DNA molecule has been reported to have a K_d_ of 10∼50 nM, and that the interactions will lead to the formation of contour products instead of the canonical nucleosome [49]. It has been reported that Nap1 is able to compete for the H2A/H2B dimer and then forms a stable Nap1-H2A/H2B complexes with a K_d_ of 1∼5 nM [49]. There is no significant change in DNA length for DNA molecules in response to the H2A/H2B dimer in the presence of histone chaperone under TPM setup. This observation can be explained by the fact that the non-chromosomal interaction between the H2A/HAB dimer and DNA are able to be prevented by the histone chaperone [49]. Our experiment here revealed a discreet BM step size of 12.5±4.8 nm for each nucleosome formation step. During the length dependent experiments, a stepwise decrease in BM amplitude averaging 12.7±5.4 nm was determined for DNA molecules with different length in response to the histone octamer (S1 Fig.), which is similar to that obtained for DNA molecules in response to H3/H4 tetramer (BM step size of 11.8±1.4 nm). The formation of the tetrasome has been shown to be the rate limiting step in the nucleosome assembly process [41]. Moreover, it has been pointed out that the formation of the hexasome via a transient tetrasome has been reported to be a quick process [8]. Therefore, the BM step size of 12.5±4.8 nm must represent the formation of tetrasome, the first step in nucleosome formation. In contrast, previous single molecule studies of the nucleosome assembly process have reported a two-step shortening in DNA length of ∼27±8 nm for the formation of tetrasome, which was followed by assembly of the H2A/H2B dimer under a force of 1 pN [43]. The possible explanations for this discrepancy include the addition of the H2A/H2B dimer being too fast to be resolved without an applied force and the change in BM amplitude corresponding to the assembly of H2A/H2B dimer being too small to be identified. Moreover, a one-step discreet shortening in DNA length of ∼50 nm has also been reported for the formation of the nucleosome on a short DNA molecules (< 8 kb) that contain tandem repeat sequences under a force of 0.3 pN using magnetic tweezers [42]. According to the structural analysis, a nucleosome is composed of a histone octamer and a 147-*bp* DNA molecule [1]. A ∼70-*bp* DNA molecule will wrap around a H3/H4 tetramer in one turn to form the tetrasome, causing significant change in length and topology. The following assembly of H2A/H2B will wrap ∼70-*bp* DNA segment in order to fasten the nucleosome core particle. However, it has been reported that ∼70-80-bp of DNA molecule are spontaneously unwrapped around the histone core [62,63]. Even though the topology will also change after the addition of H2A/H2B dimer, there is no histone H1 to tight the nucleosome structure in our system. Moreover, the resolution of TPM under our system is around 85-*bp* for 836-*bp* DNA molecule [38,56]. Therefore, the further assembly of H2A/H2B could be too dynamic and too small to be accurately determined by using TPM. Even though our TPM do not have the resolution to sense the formation of the hexasome or nucleosome, the formation of the tetrasome can be determined accurately by this experimental setup.

The DNA molecules used in our experiments were obtained by PCR using pBR322 as template, thus they do not contain any special sequence or sequences that are known to have a high affinity toward histones, such as DNA601[64]. The assembly times for nucleosome formation in the presence of Nap1 are 28.4±0.8 s, 38.5±0.7 s, 48.7±0.1 s and 61.4±0.7 s for steps I–IV respectively, indicating that the assembly rate is getting slower and slower (Fig. 2 and Table 1). Previous studies have shown that torsional stress and positive supercoiling accumulation occur during nucleosome formation, which might have an inhibiting effect on the later steps of nucleosome assembly[42]. It has also been pointed out that not only the concentration of histone, but also the force applied to the DNA molecule, will affect the nucleosome assembly rate[43,65–67]. The DNA molecules used in our system were free to swivel around the tether point, and there is no extra magnetic or optical force was applied, thus it is expected that any torsional stress will dissipate through free rotation. However, the number of potential binding sites becomes fewer based on a simple collision model, once nucleosomes have begun to occupy part of the DNA molecule. Thus it is expected that the assembly rate will become slower and slower, which also offers an alternative explanation for slower assembly rate observed here.

Histone chaperones are likely to mediate the nucleosome assembly process in two opposed ways. Firstly, they are able to temporally bind to histone molecules in order to promote or to prevent the formation H3/H4 tetramers and H2A/H2B dimers. Secondly, they are able to release free histone components allowing them to deposit on DNA molecules[68]. The assembly time was determined from the dwell time in individual status as depicted in Fig. 1A (I–IV), which corresponds to the formation of nucleosomes. It has been reported that Nap1 and Asf1 are able to form homodimers in solution and that only the dimer form will interact with either the H3/H4 tetramer or the H2A/H2B dimer to facilitate histone deposition on DNA molecules[51,61]. The high histone chaperone to histone ratio used in our system ensures that the decrease in BM amplitude and the shortening in DNA length reflect the formation of the nucleosome instead of random precipitation. Not only the nucleosome assembly rate, but also the nucleosome formation fraction within a 15 minutes reaction time, decreased in the presence of the two histone chaperones, independently. It confirms that histone chaperones are able to facilitate nucleosome assembly through temporally trapping histones, which then promotes the correct formation of the H3/H4 tetramer and the H2A/H2B dimer; this will also prevent any rapid non-nucleosomal interactions taking place. On inspection, it can be seen that Asf1 has a greater influence on the nucleosome assembly than Nap1 (Fig. 3 and S4A Fig.). It has been reported that Nap1 plays a role in the transportation of the H3/H4 tetramers and H2A/H2B dimers, especially the nuclear import and deposition of the H2A/H2B dimer[49,60]. However, it has recently been pointed out that Asf1 is able to trap most non-chromosomal complexes prior to nucleosome assembly, especially the H3/H4 tetramer [51,69]. Moreover, Asf1 is also able to bind to the H3/H4 dimer in order to prevent the formation of the H3/H4 tetramer, which highlights its role during the nucleosome assembly process[51]. Therefore, Asf1 seems to play a more crucial role in the first step of nucleosome formation than Nap1 [20,51], based on our observations.

Since histone chaperones possess highly acidic residues on their surfaces, it has been believed that charge-neutralization is their dominant mode of action to alleviate charge repulsion and eliminate non-nucleosomal histone-DNA interactions [11,49]. However, this type of regulation lacks substrate specificity. Specific hydrophobic interactions have been proposed based on co-structures as alternative histone binding modes [20,70–72]. However, it is still unclear what is the interaction mode used by histone chaperones to regulate nucleosome assembly. *In vitro* it has been reported that nucleosome reconstitution can be achieved with the aid of negatively-charged macromolecules that neutralize electrostatic interactions and prevent non-chromosomal aggregations [68]. Even though a decreased assembly rate was also observed in the presence of PGA, as well as Asf1 and Nap1, a larger decrease in BM amplitude of 19.6±3.4 nm was noted with PGA, reflecting a greater shortening of the DNA’s length (>147 bp) or alternatively the formation of non-chromosomal complexes (S3 Fig.). This implies that, although PGA is able to alleviate the histone-DNA interactions, the effect is not specific enough to facilitate the formation of nucleosomal complexes under our experimental conditions. Combining these TPM results, these findings reinforce the hypothesis that histone chaperones not only use electrostatic interactions but also use substrate-specific interactions during the nucleosome assembly process.

The fact that Nap1 is able to increase both the assembly rate and nucleosome formation fraction has been reported previously by Gaudeline *et. al.* using single molecule fluorescence videomicroscopy [41]. Experimentally, the histone concentration is almost the same between these two sets of experiments; nevertheless, our study revealed a slower nucleosome assembly rate in the presence of histone chaperones. This discrepancy can be explained based on the following experimental differences. Firstly, the histones were mixed with twice the amount of histone chaperone at 30°C for 15 minutes rather than room temperature before initiation of nucleosome assembly in our experimental setup. Secondly, a lower concentration of histone chaperone was used under Gaudeline’s experiment setup, which may have led to the presence of non-nucleosomal complexes. Thirdly, the DNA molecules used in our system are shorter than 1 kb B-form relaxed DNA compared to λ-dimer DNA stained with YOYO-1 iodide used in Gaudeline’s experiment setup; specifically it is unclear whether YOYO-1 staining has any influence on DNA topology. Finally, we used 150mM NaCl rather than 50mM NaCl and it has been known that the ionic strength will affect the histone-histone chaperone-DNA interactions significantly. In such circumstances, the differences in experimental results are perhaps not unexpected. The same experiments were performed under 50mM NaCl, a slower nucleosome assembly was observed. Moreover, the nucleosome formation fraction obtained within a 15 minutes reaction time was smaller in the absence of histone chaperone (S5B Fig.). These findings suggest that histone components easily decompose under lower ionic strength condition, while histone chaperone plays a role to facilitate the formation of H3/H4 tetramer and H2A/H2B dimer for the nucloesome formation, consistent with Gaudeline’s experimental results. Therefore, the main reason for the experimental inconsistence is the ionic strength effect.

**Figure 5:**
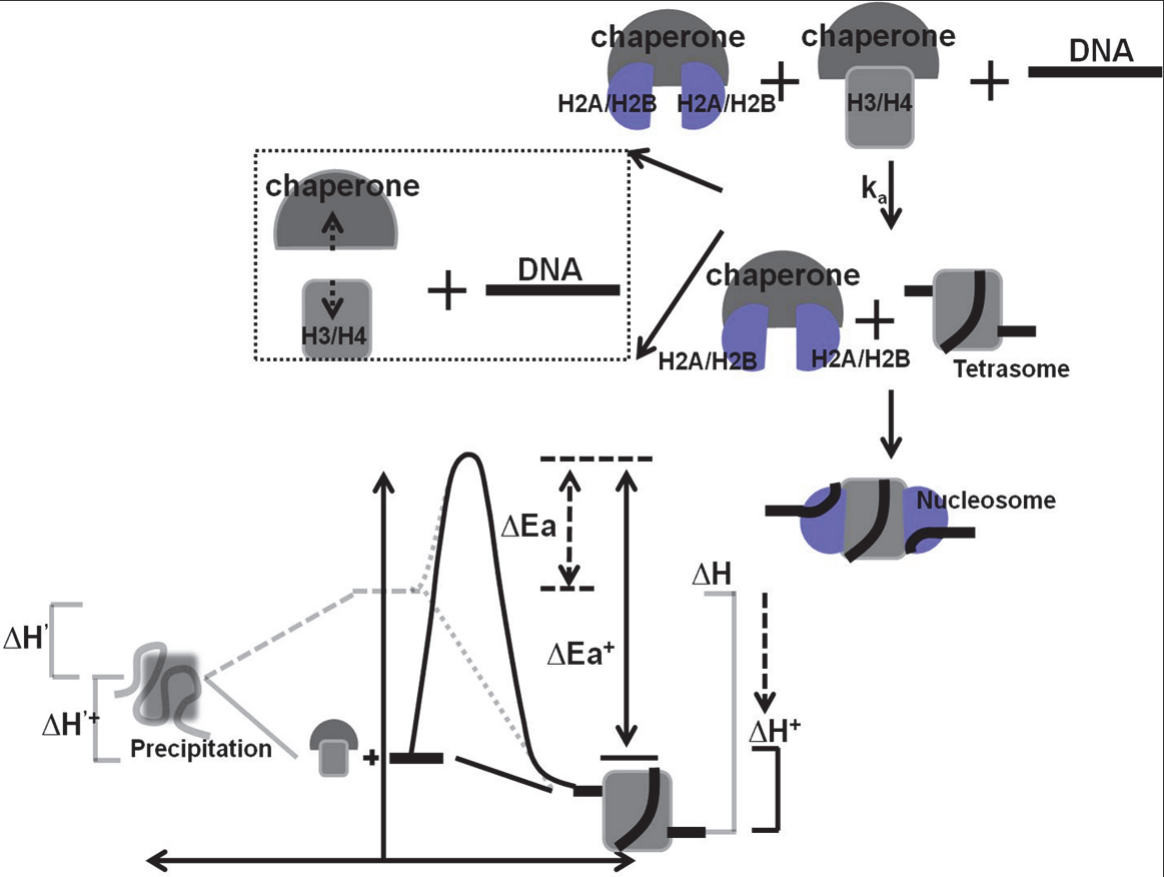
Model outlining the histone chaperone-mediated nucleosome assembly process illustrated in free-energy reaction diagram. Only the nucleosome assembly process is chosen for analysis using tethered particle motion experiment. The discreet decrease in the BM amplitude signals the formation of tetrasome, which is known to be the rate limited step of nucleosome formation, and this is represented by k_a_. The proposed reaction scheme is plotted showing reactant (DNA and histone, middle) and product (precipitation, left, or tetrasome, right). Based on our findings, it would seem that histone chaperone stabilizes the histones and facilitates the formation of enthalpy-favored nucleosomal complexes with free histones as reaction substrates.

Two models have been proposed to describe histone chaperone-mediated nucleosome assembly based on the thermodynamic parameters obtained in various studies. In the first model, histone chaperones stabilize the histone and make the formation of nucleosome complexes energetically favorable compared to non-nucleosomal complexes [8,11,49]. However, it is unclear whether the substrates in the nucleosome assembly process are the histone-histone chaperone complexes or the free histones in dynamic equilibrium. In second model, histone chaperones mediate the formation of ternary transition complexes, namely histone-histone chaperone-DNA. This allows a new reaction pathway with lower activation energy to be available that leads to the nucleosome formation in favor of non-nucleosomal complexes [11,73]. By using single-molecule TPM to investigate the histone chaperone-mediated nucleosome assembly process, a potential mechanism of histone chaperones can be put forward in the form of a simplified free-energy reaction diagram and based on the above kinetic results (Fig. 5). In our study, compared to the kinetic behavior in the absence of histone chaperone, a slower assembly rate was found. This finding suggests that histone chaperones stabilize histones and facilitate the formation of thermodynamically-favored products via tetrasome or hexasome as intermediates along a reaction path with higher activation energy. Based on our proposed model, the assembly rate will depend on the concentration of histone and histone chaperone if the reaction substrate of nucleosome assembly process is free histones in dynamic equilibrium. On the other hand, the assembly rate will remain the same during the concentration manipulation if histone chaperone-histone complexes are the reaction substrates for acceptor DNA molecules. Based on the results of the concentration dependent experiments, a slower assembly rate and a smaller nucleosome formation fraction were obtained as the concentration of histone chaperone was raised. This supports the hypothesis that free histones in dynamic equilibrium rather than chaperone-histone complexes are the reaction substrate during the nucleosome assembly process. It is known that free histones inside cells are rare or completely absent and most purified H3/H4 tetramer proteins obtained from cell extracts have been found to be chaperone-bound[74]. Moreover, it has been reported that Nap1 binds to the H2A/H2B dimer with a high affinity of 7.8±0.4 nM and prevents non-canonical interaction between the H2A/H2B dimer and DNA (K_d_ ∼44 nM). Therefore, a histone chaperone with a high histone binding affinity will facilitate nucleosome formation by disfavoring non-nucleosomal interactions [49], and this is consistent with our proposed model. Taken all together, our kinetic findings support a model wherein histone chaperones will stabilize histones and facilitate the formation of the enthalpy-favored nucleosome with free histone in dynamic equilibrium as reaction substrate in vitro. In the future, more experiments are required to verify the functional roles of histone chaperones in nucleosome structure regulation.

## Materials and Methods

### Proteins and DNA substrates

Histone octamers were purchased from Abcam (ab45275) and purified by gel filtration (GE, S200). The expression plasmids yNap1 and yAsf1 were gifts from Bradley R. Carins (University of Utah) and Jessica K. Tyler (University of Texas) respectively. yNap1 (Nap1) was expressed and purified by Ni-NTA column and ion-exchange chromatography [50]. yAsf1 (Asf1) was expressed and purified by GST column and ion-exchange chromatography, and the GST tag was removed using thrombin digestion (Sigma) [51]. All the DNA molecules (211-*bp*, 433-*bp*, 614-*bp* and 836-*bp*) were prepared by PCR using pBR322 (NEB) as the template. The primer sequences are listed in Table 2.

**Table 2:** Primer sequences used to obtain the experimental DNA sequences.

**DNA substrate**	**Template**	**Primer sequence**
836-bp	pBR322	DigN –CGTCACCCTGGATGCTGTAG Bio– CGTAGCCCAGCGCGTCG
614-bp	pBR322	DigN –GTGGAACGAAAACTCACG Bio– TGAGTGATAACACTGCGGC
433-bp	pBR322	DigN –CGTCACCCTGGATGCTGTAG Bio– GATGGCGCCCAACAGTCCC
211-bp	pBR322	DigN –CGTCACCCTGGATGCTGTAG Bio– GGCTCCAAGTAGCGAAGCG

### Single-molecule TPM measurement and data analysis

The reaction chambers were prepared as previously described [37,38,52–56], and all experiments were performed under an inverted optical microscope (IX-71, Olympus, 100x objective with NA = 1.40) using a differential interference contrast (DIC) imaging system at 22°C. An analog camera (Newvicon-70, frame interval: 33ms) was used to acquire the experimental images, while the illumination light source was replaced with a mercury lamp in order to enhance the image contrast. The data analysis and criteria followed the same procedures as previously described [37,52,53]. All the Brownian motion (BM) data were smoothed using five-point adjacent averaging. Only those molecules with a BM amplitude within the 95% confidence level using a 40-frame averaging window before initiation of reaction were chosen as bare DNA molecules, and then used for data analysis. Molecules that stuck to the glass surface (with BM <7 nm) for more than five points or exhibited distorted movement (the ratio of BM in the × coordinate to BM in the y coordinate being outside the range of 0.8 to 1.2) at any time were excluded.

The dwell time distribution histogram was fitted to a single exponential decay algorithm with following formula:

$$y=A_{1}\times e^{-k_{1}t}$$

where A_1_ and k1 are the fitting parameters determined by Origin 8.0.

All DNA molecules were labeled with digoxigenin and biotin at their 5′ ends, and immobilized on the coverglass surface by digoxigenin-anti-digoxigenin interactions. Next the other end of each DNA molecule was labeled with a streptavidin-coated 200 nm polystyrene bead, which acted as the reporter. Nucleosome assembly was initiated by adding complete reaction solution containing 7.5 nM histone (H2A/H2B dimer, H3/H4 tetramer or octamer) in the presence of a specific concentration of histone chaperone (Nap1 or Asf1) or poly glutamate acid (PGA) prepared in reaction buffer (10 mM Tris-HCl, 0.5 mM EDTA, 150 mM NaCl, pH = 8.0, 5 mM dithiothreitol and 1 mg/mL BSA). In order to further verify the chaperone effect, the same experimental procedure was performed under low salt condition (10 mM Tris-HCl, 0.5 mM EDTA, 50 mM NaCl, pH = 8.0, 5 mM dithiothreitol and 1 mg/mL BSA). The complete reaction solution was mixed and incubated at 30°C for 15 minutes before initiation of experiments. All experiments were performed at room temperature (22°C).

## Supporting Information

S1 Fig The representative time trace and the Brownian motion amplitude distribution for the tethered particle motion experiment investigating the histone chaperone-mediated nucleosome assembly process. (A)–(B) 211-*bp* DNA in response to the addition of histone octamer along with Nap1. A step size of 12.6±0.3 nm was determined. (C)–(D) 433-*bp* DNA in response to the addition of histone octamer along with Nap1. Step sizes of 12.2±3.6 nm and 13.4±3.6 nm were determined. (E)–(F) 614-*bp* DNA in response to the addition of histone octamer along with Nap1. Step sizes of 13.1±1.0 nm, 12.1±1.1 nm, and 13.0±0.9 nm were determined. The hatched bar represents the expected BM amplitude for the specific length DNA molecules in response to the addition of histone. The black line indicates the initiation of reaction. The assembly trace presents one, two and three-step shortening of the DNA length for the 211-*bp*, 433-*bp* and 614-*bp* DNA molecules respectively.
(TIF)

S2 Fig Tethered particle motion experiment investigating the histone chaperone-mediated nucleosome assembly process. The representative time trace for a 836-*bp* DNA molecule in response to the addition of (A) 15nM Asf1 only. (B) 37.5nM PGA only. The black line indicates the initiation of reaction.
(TIF)

S3 Fig The change in Brownian motion amplitude during each individual assembly step. The histograms were constructed from the pooled Brownian motion amplitude changes during the assembly state as illustrated in Fig. 1A. (A) 7.5 nM Histone only. (B) 7.5 nM Histone along with 15 nM Nap1. (C) 7.5 nM Histone along with 15 nM Asf1. (D) 7.5 nM Histone along with 37.5 nM PGA.
(TIF)

S4 Fig Kinetic analysis of the dwell times during the first step for 836-bp DNA molecules in response to histone octamer under 50mM NaCl. The histograms were constructed from the pooled distribution of the dwell times during the assembly state as illustrated in Fig. 1A Step I. (A) (1) 7.5 nM Histone (N = 34). (2) 7.5 nM Histone along with 15 nM Nap1 (N = 40). (3) 7.5 nM Histone along with 15 nM Asf1 (N = 26). (B) The distribution of BM amplitude for 836-*bp* DNA molecules in response to the addition of histone octamer (1) (N = 77) or along with (2) 15 nM Nap1 (N = 85) (3) 15 nM Asf1 (N = 41). All the data were fitted using a single-exponential decay algorithm written in Origin 8.0.
(TIF)

S5 Fig Kinetic analysis of the dwell times during the individual steps for 836-bp DNA molecules in response to histone octamer. The histograms were constructed from the pooled distribution of the dwell times during the assembly state as illustrated in Fig. 1A. (A) 7.5 nM Histone along with 37.5 nM Asf1, (1)–(4) represent the pooled dwell time for Step I–IV respectively. (B) 7.5 nM Histone along with 70 nM Asf1, (1)–(2) represent the pooled dwell time for Step I–II respectively.. All the data were fitted using a single-exponential decay algorithm written in Origin 8.0 and are listed in Table 2.
(TIF)
