# Supplementary figures and images for: Histone Chaperone-Mediated Nucleosome Assembly Process
Source: PLoS One. 2015 Jan 22;10(1):e0115007. doi: 10.1371/journal.pone.0115007 (PMC4303269; doi:10.1371/journal.pone.0115007)

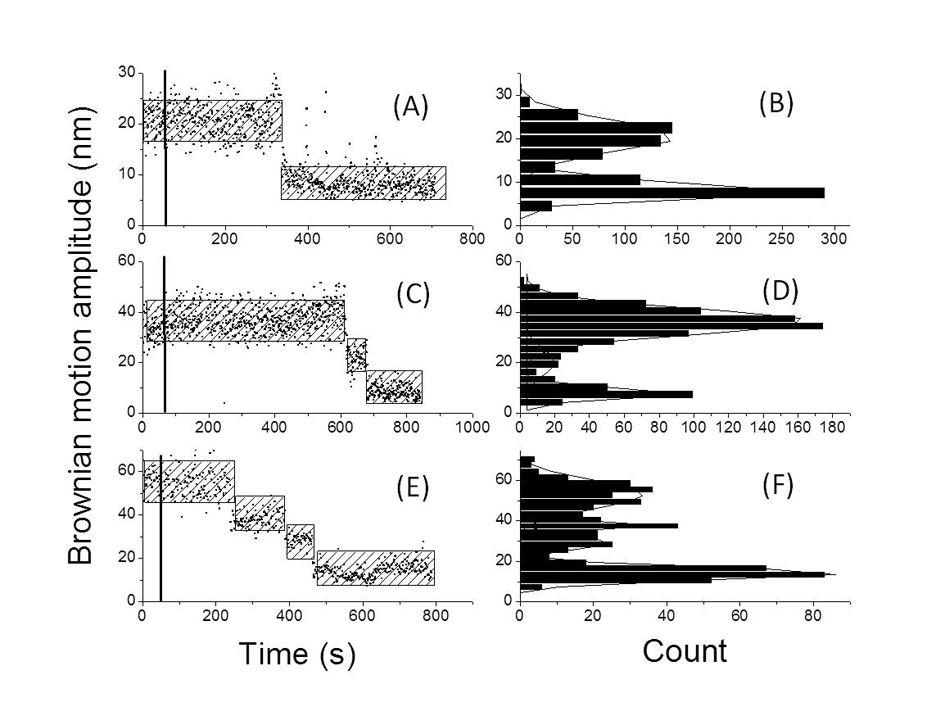

Supplement: S1 Fig — (A)–(B) 211-bp DNA in response to the addition of histone octamer along with Nap1. A step size of 12.6±0.3 nm was determined. (C)–(D) 433-bp DNA in response to the addition of histone octamer along with Nap1. Step sizes of 12.2±3.6 nm and 13.4±3.6 nm were determined. (E)–(F) 614-bp DNA in response to the addition of histone octamer along with Nap1. Step sizes of 13.1±1.0 nm, 12.1±1.1 nm, and 13.0±0.9 nm were determined. The hatched bar represents the expected BM amplitude for the specific length DNA molecules in response to the addition of histone. The black line indicates the initiation of reaction. The assembly trace presents one, two and three-step shortening of the DNA length for the 211-bp, 433-bp and 614-bp DNA molecules respectively. (TIF) [file pone.0115007.s001.tif]

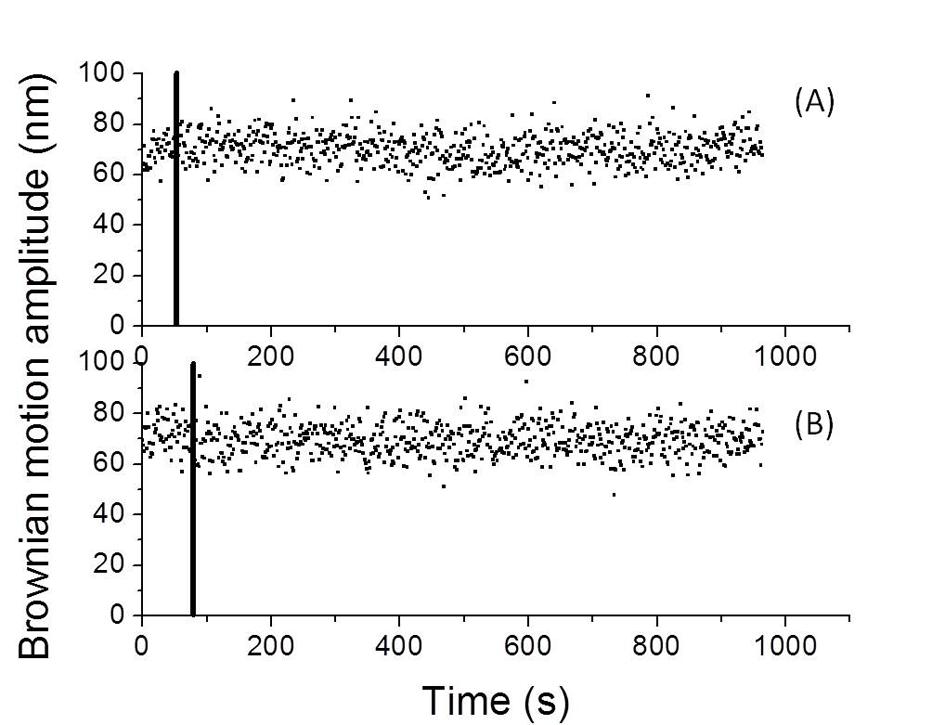

Supplement: S2 Fig — The representative time trace for a 836-bp DNA molecule in response to the addition of (A) 15nM Asf1 only. (B) 37.5nM PGA only. The black line indicates the initiation of reaction. (TIF) [file pone.0115007.s002.tif]

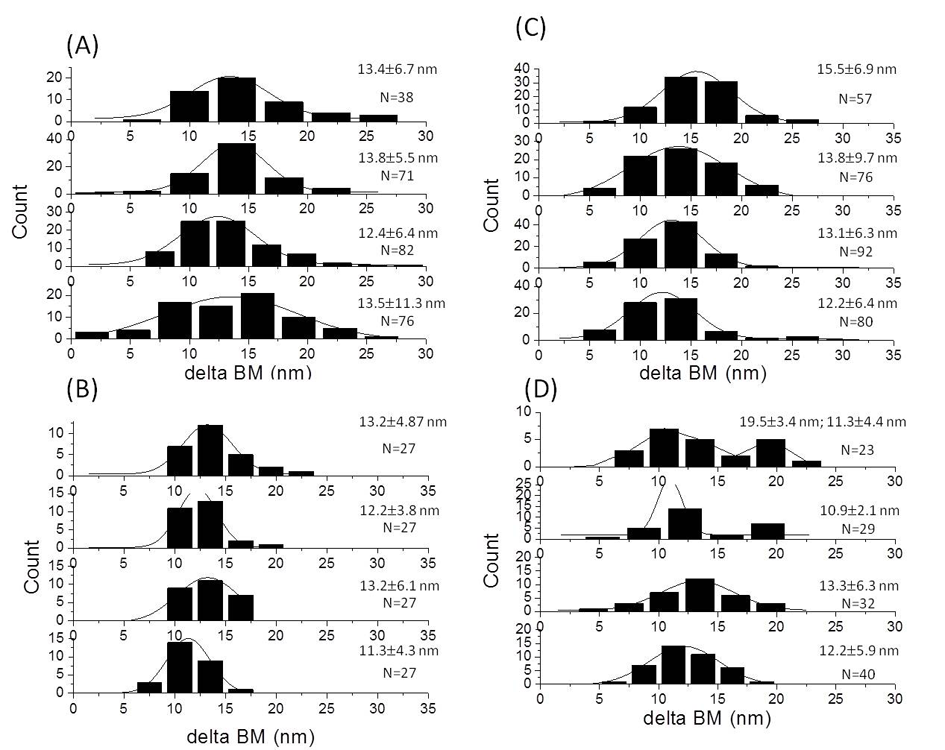

Supplement: S3 Fig — The histograms were constructed from the pooled Brownian motion amplitude changes during the assembly state as illustrated in Fig. 1A. (A) 7.5 nM Histone only. (B) 7.5 nM Histone along with 15 nM Nap1. (C) 7.5 nM Histone along with 15 nM Asf1. (D) 7.5 nM Histone along with 37.5 nM PGA. (TIF) [file pone.0115007.s003.tif]

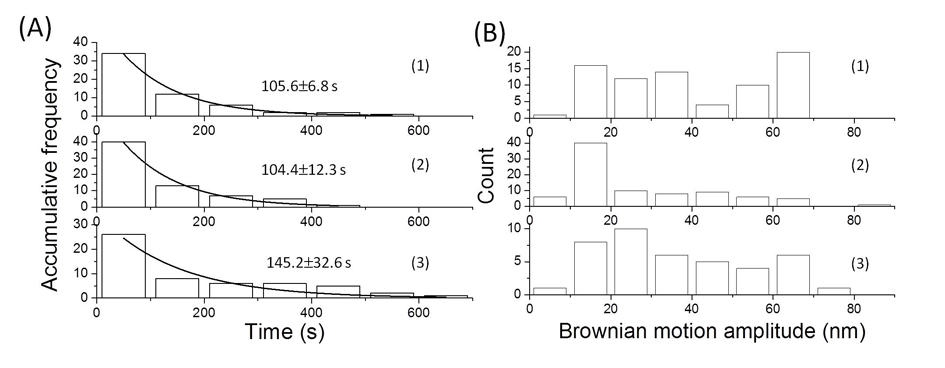

Supplement: S4 Fig — The histograms were constructed from the pooled distribution of the dwell times during the assembly state as illustrated in Fig. 1A Step I. (A) (1) 7.5 nM Histone (N = 34). (2) 7.5 nM Histone along with 15 nM Nap1 (N = 40). (3) 7.5 nM Histone along with 15 nM Asf1 (N = 26). (B) The distribution of BM amplitude for 836-bp DNA molecules in response to the addition of histone octamer (1) (N = 77) or along with (2) 15 nM Nap1 (N = 85) (3) 15 nM Asf1 (N = 41). All the data were fitted using a single-exponential decay algorithm written in Origin 8.0. (TIF) [file pone.0115007.s004.tif]

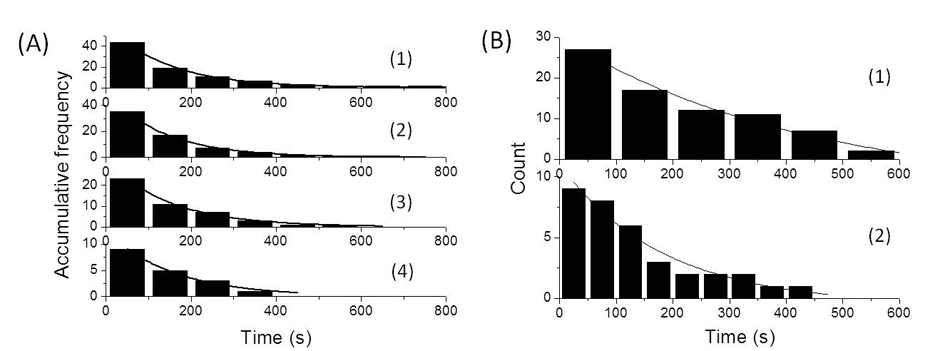

Supplement: S5 Fig — The histograms were constructed from the pooled distribution of the dwell times during the assembly state as illustrated in Fig. 1A. (A) 7.5 nM Histone along with 37.5 nM Asf1, (1)–(4) represent the pooled dwell time for Step I–IV respectively. (B) 7.5 nM Histone along with 70 nM Asf1, (1)–(2) represent the pooled dwell time for Step I–II respectively.. All the data were fitted using a single-exponential decay algorithm written in Origin 8.0 and are listed in Table 2. (TIF) [file pone.0115007.s005.tif]
